# Rapid fixation and immunofluorescent staining of cultured cells using microwave irradiation

**DOI:** 10.1179/014788811X12949268295960

**Published:** 2011-03

**Authors:** Kazuo Katoh

**Affiliations:** Department of Anatomy, Jichi Medical University, Shimotsuke-city, Tochigi, Japan

**Keywords:** Cell Culture, Immunofluorescence microscopy, Immunostaining, Microwave

## Abstract

Microwave irradiation during tissue fixation and immunostaining reduces the sample preparation time. Microwave irradiation also facilitates the penetration of fixatives and antibody solutions into the tissues, resulting in efficient fixation and reduction of non-specific antibody binding, respectively. Experimental procedures involving immunofluorescence microscopy are time-consuming as this method relies on antigenantibody reaction. Here, we utilized a technique involving exposure of cultured cells and tissues to intermittent microwave irradiation and immunostaining of fixed samples. Intermittent microwave irradiation during fixation reduces the required incubation time with blocking and antibody solutions, and results in good preservation of the immunoreactivity of fixed cells. Microwave irradiation also reduces the non-specific binding of fluorescein-labeled antibodies. These microwave-assisted rapid immunofluorescence techniques are useful for analysis of molecular compositions in cultured cell systems.

## Introduction

Immunofluorescence microscopy techniques are powerful methods for research in a wide range of areas, especially in pathological studies. Indirect immunofluorescence techniques are commonly used in routine laboratory studies. Immunofluorescence microscopy is used to detect certain types of protein in tissues based on antigen–antibody reactions. The specimens are first reacted with unlabeled primary antibody, and then a secondary antibody directed against the immunoglobulin type of the primary antibody is produced and conjugated to a fluorochrome. Although immunofluorescence microscopy has been in use for over 25 years, the method has remained relatively unchanged. As immunofluorescence staining makes use of antibody–protein reaction, it takes several hours to complete the fixation and immunofluorescence staining procedures. General immunofluorescence procedures involve several antibody incubation steps, each of which may take 1 or 2 h. Long antibody incubation times, especially for fluorescein-labeled secondary antibodies, seem to result in increased non-specific binding. As immunofluorescence microscopy can be used in combination with other non-antibody markers, such as DAPI for nuclei staining, fluorescein-labeled phalloidin for F-actin staining, fluorescent analogs for staining of the Golgi apparatus, endoplasmic reticulum, mitochondria, etc., additional time is also required to process the samples. For immunofluorescence microscopy, the microwave irradiation technique significantly reduces the incubation times with both antibodies and the number of washes/and the time for washing with buffer solution.

Microwave irradiation during tissue processing reduces the processing times required for chemical fixation with paraformaldehyde, decalcification of bone tissues, staining with chemical reagents, and retrieval of antibody antigen interactions on paraffin-embedded tissues.^1^–^8^ Previous studies have also demonstrated that microwave irradiation significantly reduces the incubation times with primary and secondary antibodies in immunofluorescence microscopy experiments.^9^–^12^ Microwave irradiation of paraffin-embedded tissues is also utilized for fluorescence *in situ* hybridization analysis.^13^ Recent advances in microwave irradiation devices allow the control of irradiation power, irradiation time, and intermittent microwave irradiation.^12^,^14^–^16^ Therefore, using modern equipment, microwave irradiation is expected to become applicable to many types of histological technique. Intermittent microwave irradiation during tissue fixation reduces the incubation time with fixative resulting in better preservation of tissue morphology. Microwave irradiation during the immunolabeling of tissues significantly reduces the incubation time with antibody solution, thus reducing non-specific antibody binding and minimizing background noise, which is a major drawback of immunofluorescence microscopy. Microwave-assisted fixation and immunofluorescence staining have many advantages for examination of cultured cell systems *in vitro*.

Cultured cell systems, such as fibroblastic, endothelial, brain, and embryonic cells, are powerful models for use in cellular and molecular biology studies. Immunofluorescence microscopy and cultured cell systems are essential tools for fundamental pathological and molecular research. As mentioned above, immunofluorescence microscopy is time-consuming because it makes use of antigen–antibody reactions. It is important to reduce the times required for fixation, immunostaining, and washing to increase the efficiency of thee methods.

Here, we report a rapid procedure for both fixation and immunostaining of cultured cell systems, such as fibroblastic and endothelial cells. The incubation times required for fixation, with blocking solution, and with antibody solution, have been markedly reduced by microwave irradiation of the samples. In addition, non-specific binding of antibodies was also markedly reduced. This rapid immunofluorescence method will prove useful for analysis of the molecular composition and function of many cultured cell systems, including fibroblastic cells, central nervous system cells, the tissues of various organs, etc.

## Materials and Methods

### Antibodies and fluorescent reagents

Monoclonal anti-actin (Sigma, St Louis, MO, USA), anti-vinculin (Sigma), anti-alpha-actinin (Abcam, Cambridge, MA, USA), and anti-talin (BD Transduction Laboratories, San Jose, CA, USA) were purchased from the sources indicated. Polyclonal fluorescein (FITC)-labeled goat anti-mouse IgG was purchased from Cappel (Durham, NC, USA) and used as the secondary antibody.

### Cell culture

**Figure 1:**
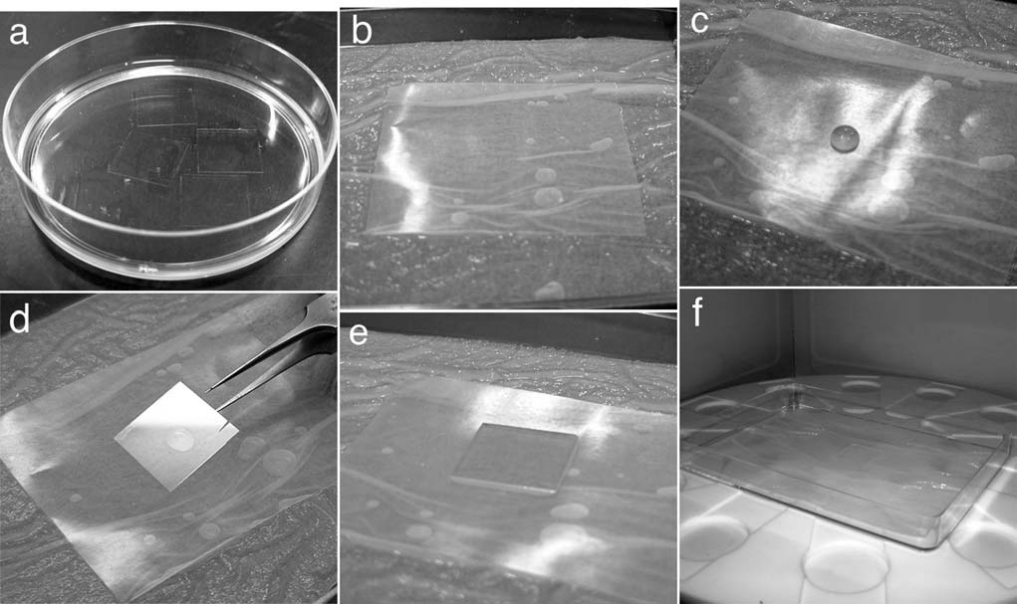
Materials and set-up of immunofluorescence microscopy with microwave irradiation. Cultured cells on coverslips measuring 18×18 mm were incubated with a DMEM/F12 culture medium (a). A drop (50 *μ*l) of diluted blocking solution or antibody solution was dispensed onto the surface of a piece of wax film in the wet chamber (c). Cells were then placed upside down on the drop of blocking solution for 5 min (d and e) and placed in the microwave oven (f).

**Figure 2:**
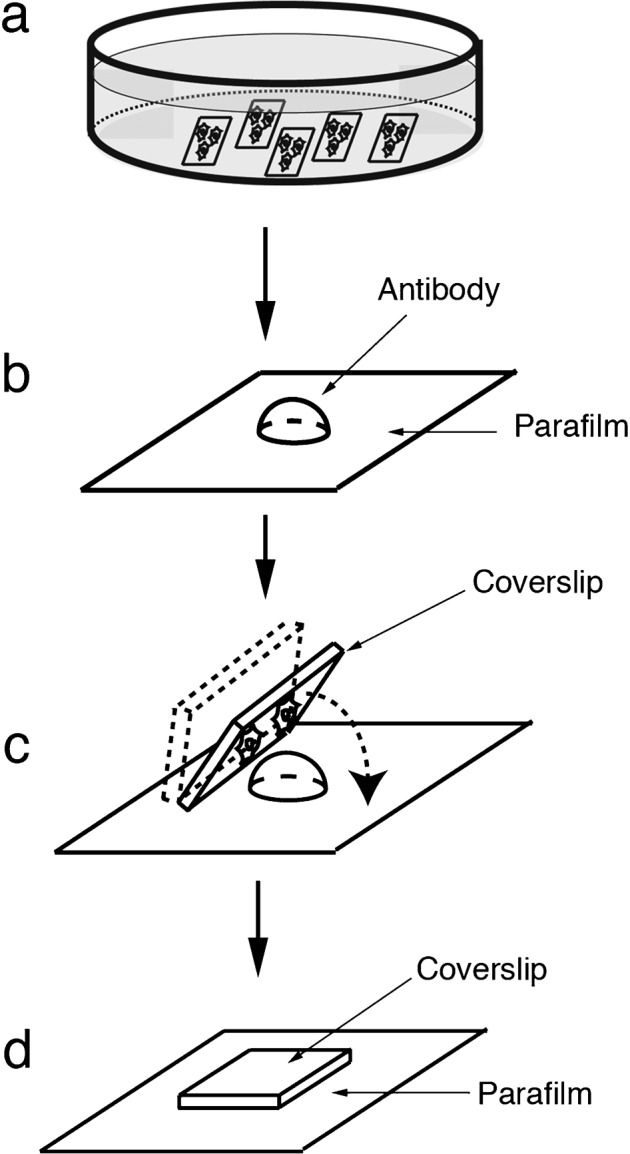
Schematic illustrations of incubation methods with blocking solution or antibody solution on wax film. A 50-*μ*l drop of diluted blocking solution or antibody solution was dispensed onto the surface of a piece of wax film in the wet chamber (b), followed by gently coverslipping the cells upside down on the drop (d), and transfer to the microwave oven (d).

Human foreskin fibroblasts (FS-133) and bovine endothelial cells were cultured on coverslips (22×22 or 18×18 mm; Matsunami, Tokyo, Japan) in culture dishes (*φ*100×20 mm height; Falcon Plastics, Los Angeles, CA, USA) (Figs. 1a and 2a) with a 1∶1 mixture of Dulbecco’s modified Eagle’s medium (DMEM) and a nutrient mixture consisting of F-12 (Gibco, Grand Island, NY, USA), pH 7·4, containing 50 U/ml penicillin, 50 μg/ml streptomycin, and 10% fetal bovine serum (Salmond Smith Biolab, Aukland, New Zealand). The cells were maintained at 37°C in a humidified atmosphere of 5% CO_2_ overnight.

### Immunofluorescence microscopy with microwave irradiation

In this study, a microwave oven was used to apply intermittent microwave irradiation to the samples (microwave oven equipped for laboratory use; Azumaya MI-77; Nippon Automatic Control Company, Tokyo, Japan). For fixation, cells on culture dishes were washed briefly with three changes of phosphate buffered saline (0·1 M; PBS). The medium was replaced with fixative (1% paraformaldehyde in PBS), followed immediately by application of intermittent microwave irradiation for 5 minutes (4 second ON, 3 second OFF; 200–250 W) (Table 1). The fixed samples were then rinsed three times with PBS for 5 minutes each time without microwave treatment.

**Table 1:** Protocol of immunofluorescence microscopy with intermittent microwave irradiation

1. Culture cells on coverslips according to standard procedures
2. Wash cells briefly with three changes of PBS
3. Fix cells in paraformaldehyde with intermittent microwave irradiation (5 minutes; 4 second ON, 3 second OFF)
4. Rinse fixed cells three times in PBS for 5 minutes each time without microwave irradiation
5. Incubate cells with Triton X-100 in PBS for 5 minutes without microwave irradiation
6. Wash briefly with three changes of PBS
7. Incubate samples with blocking solution for 5 minutes with microwave irradiation
8. Wash samples briefly with three changes of PBS
9. Incubate samples with the primary antibody for 5 minutes with microwave irradiation
10. Wash briefly with three changes of PBS
11. Incubate samples with FITC-labeled secondary antibody for 5 minutes with microwave irradiation
12. Wash briefly with three changes of PBS
13. Mount specimen cell-side down on the slideglasses with anti-bleaching solution
14. Glue coverslip to the slideglass using nail polish
15. Observe by fluorescence microscopy

A wet chamber was made in a dish measuring 10×14 cm (Eiken, Tokyo, Japan) lined with a sheet of absorbent paper (KimWipe; Kimberly-Clark, Tokyo, Japan) with addition of water to prevent desiccation (Fig. 1b). A small drop of 50 μl of diluted blocking solution (10% normal goat serum in PBS) was dispensed onto the surface of an appropriately sized piece of wax film (Parafilm; Pechiney Plastic Packaging, Menasha, WI, USA) in the wet chamber (Figs. 1b and c and 2b).

Fixed cells were then placed upside down on the drop of blocking solution for 5 minutes (Figs. 1d and e and 2c and d) with microwave irradiation (4 second ON, 3 second OFF) (Fig. 1f). Samples were placed cell-side up in culture dishes, and briefly washed three times with PBS for 5 minutes each time without microwave treatment. They were then incubated with 50 μl of anti-actin (1∶100), anti-vinculin (1∶400), anti-alpha-actinin (1∶100) or anti-talin (1∶100) antibody, in a drop of medium on the wax film in the same way as described above for 5 minutes with microwave irradiation. The samples were again washed in PBS, incubated with FITC-labeled goat anti-mouse IgG (1∶100) for 5 minutes with microwave irradiation, and washed briefly with three changes of PBS. The samples were then mounted in a drop of mounting medium (Vectashield; Vector, Burlingame, CA, USA).

**Figure 3:**
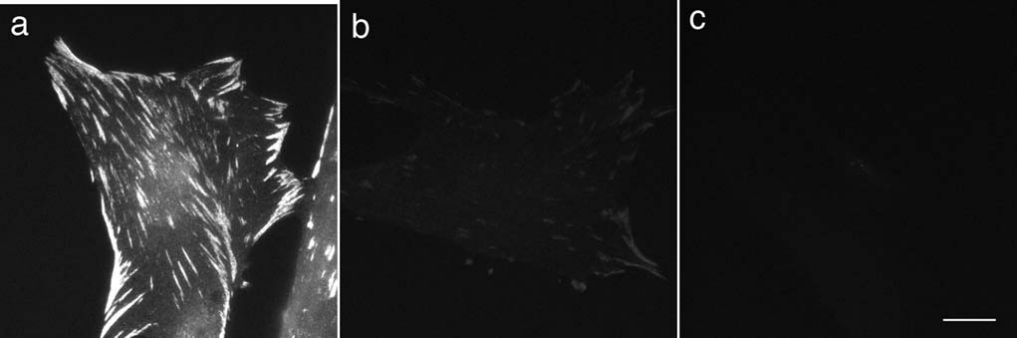
Conventional imunofluorescent microscopy stained with anti-vinculin and control experiments. Human foreskin fibroblasts were conventionally fixed and stained with anti-vinculin without microwave irradiation (a). Immunofluorescent intensity was almost the same as that of rapid staining with microwave irradiation. When fixed cells were incubated with anti-vinculin without microwave irradiation (first antibody for 5 minutes and then secondary antibody for 5 minutes), only faint immunofluorescent staining was exhibited (b). Fixed cells incubated only with fluorescent-labeled secondary antibody, without first antibody, did not show any immunofluorescent signaling (c). Bar = 20 *μ*m.

As controls, cells in culture were fixed and stained with anti-vinculin antibody (1∶400) for 5 minutes without microwave irradiation. After washing in PBS, samples were incubated with fluorescein-labeled goat anti-mouse IgG for 5 minutes without microwave irradiation, and then washed with PBS and mounted in a drop of mounting medium. These samples showed only faint immunofluorescent staining (Fig. 3b). Samples that were incubated only with fluorescently-labeled secondary antibody without first antibody did not show any fluorescent staining (Fig. 3c). Conventional immunofluorescent staining with anti-vinculin antibody (first antibody for 1 hour and secondary antibody for 1 hour without microwave) was also shown in Fig. 3a.

Samples were observed using an AxioPhot epifluorescence microscope with an Apochromat ×63 (N.A. 1·4, oil) objective lens (Carl Zeiss Inc., Oberkochen, Germany), or a BX-51 epifluorescence microscope with PlanApo X60 (N.A. 1·4, oil) objective lens (Olympus, Tokyo, Japan). The protocol used for immunofluorescence microscopy with intermittent microwave irradiation is shown in Table 1.

## Results and Discussion

Microwave irradiation during tissue processing markedly reduces the time required for fixation, decalcification, staining with chemical reagents, and incubation with antibodies. However, conventional commercially available high-output microwave ovens are unsuitable for irradiation of small tissue samples, as it is not possible to accurately control the temperature of samples using such microwaves. Therefore, conventional microwave processors are unsuitable for biomedical research. However, microwave devices specifically adapted for histochemistry have recently become commercially available. With the MI-77 microwave processor (Azumaya Co., Tokyo, Japan), it is possible to control the strength of irradiation from 150 to 400 W. It is also possible to completely regulate the temperature using two independent temperature control systems, i.e. infrared and thermocouple temperature measurement systems. General immunofluorescence procedures involve several antibody incubation steps, each of which takes between 1 and 2 hours. However, long periods of incubation with antibodies, especially fluorescein-labeled secondary antibodies, seems to increase non-specific binding. In this study, we applied intermittent microwave irradiation to cultured cell systems consisting of human foreskin fibroblasts and bovine endothelial cells.

This article outlines the methods used for the rapid immunofluorescence staining of cultured cell systems using intermittent microwave irradiation. Investigations of macromolecular composition and functional analyses generally use cultured model systems because they are more amenable to study than normal tissues *in situ*. However, immunostaining procedures are very time-consuming. As mentioned above, intermittent microwave irradiation by microwave oven, Azumaya MI-77, reduced the incubation time required to fix the cells, and thus better preserved immunostaining. The total time required for processing was 30 minutes, representing a reduction of about one-fourth in comparison to standard methods. This method will be useful for analyses of the molecular composition and function of cultured cell systems *in vitro*. This rapid immunofluorescence method is also applicable to pathological studies of cultured cancer cells, developmental analysis of embryonic stem cells, and differentiation analysis of induced pluripotent stem cells. Moreover, microwave irradiation yields low-background, high-contrast images. Microwave irradiation during immunolabeling of tissues significantly reduces the incubation time with antibody solution, and thus reduces non-specific antibody binding.

**Figure 4:**
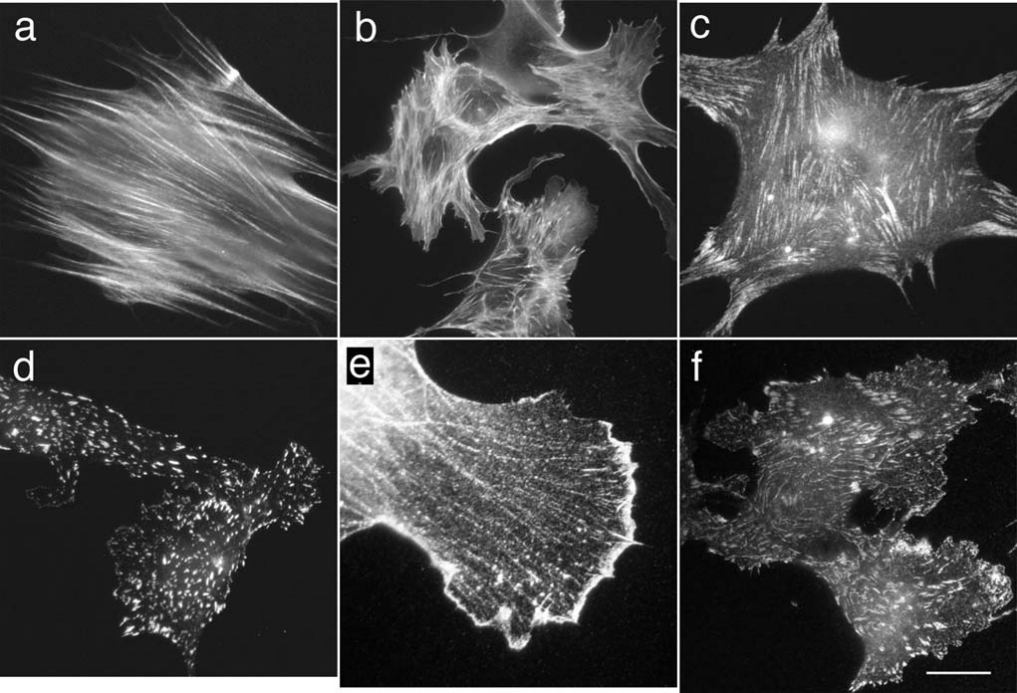
Fluorescence micrographs of microwave-assisted fixation and immunofluorescent staining. Fixed human foreskin fibroblasts (a, c, and e) and bovine endothelial cells (b, d, and f) were stained with anti-actin (a and b), anti-vinculin (c and d), anti-alpha-actinin (e), or anti-talin (f) antibody for 5 minutes, and then with fluorescent-labeled secondary antibodies for 5 minutes with intermittent microwave irradiation. In both fibroblasts and endothelial cells, immunoreactivity with anti-actin antibody was observed along the actin-containing microfilament bundles called stress fibers (a and b). Anti-vinculin staining was observed in a punctate pattern, reflecting cell-to-substrate adhesion sites in both fibroblastic and endothelial cells (c and d). Anti-alpha-actinin staining was colocalized with stress fibers (e). Anti-talin staining was also localized to focal adhesion sites in bovine endothelial cells (f). Bar = 20 *μ*m.

In this study, we utilized microwave irradiation with immunofluorescence microscopy for cytoskeletal protein markers to detect actin, alpha-actinin, vinculin, and talin (Fig. 4). All of the antibodies were directed against typical cytoskeletal proteins in the cultured cells. In both fibroblasts and endothelial cells, immunoreactivity with anti-actin antibody was observed along the actin-containing microfilament bundles called stress fibers (Fig. 4a and b). Staining for alpha-actinin, which is known to be colocalized with stress fibers, was also clearly seen with microwave irradiation in fibroblasts (Fig. 4e). Immunostaining with anti-vinculin antibody, which reflects cell-to-substrate adhesion sites at the ends of stress fibers called focal adhesion, is observed in a punctate pattern (Fig. 4c and d). Immunostaining for talin, which is known to be localized at the focal adhesion sites, was also detected correctly with microwave irradiation. As control, cells were conventionally fixed and stained with anti-vinculin antibody for 1 hour, then with fluorescein-labeled secondary antibody for 1 hour, showed the same staining intensity compared with microwave-assisted procedure (Fig. 3a for conventional immunofluorescent staining and Fig. 4c for microwave assisted procedure). As another controls, cells were fixed and stained with anti-actin, anti-vinculin, anti-alpha-actinin, and anti-talin antibodies for 5 minutes without microwave irradiation. The above samples exhibited only faint immunofluorescence staining (see Fig. 3b for anti-vinculin staining). The samples incubated only with secondary antibodies did not exhibit any immunofluorescent staining, indicating that there was no non-specific binding of secondary antibody (Fig. 3c).

## Conclusions

Intermittent microwave irradiation during tissue fixation reduced the incubation time in fixative, which resulted in good preservation of tissue morphology. Microwave irradiation during immunolabeling of tissues significantly reduces the incubation time with antibody solution, thus reducing non-specific antibody binding and consequently minimizing background noise. Microwave-assisted fixation and immunofluorescence staining showed many advantages for the examination of cultured cell systems *in vitro*.
